# A neuroimmune-enriched multibiofluid proteomic index associated with cognitive-motor progression in early Parkinson’s disease: a longitudinal PPMI cohort study

**DOI:** 10.3389/fimmu.2026.1928940

**Published:** 2026-09-14

**Authors:** Hao Wang, Guoqing Wu, Dengke Zhang, Wanjun Feng, Xuebin Zhao, Weixiong Zhang, Ganqin Du

**Affiliations:** 1 The First Affiliated Hospital of Henan University of Science and Technology, Luoyang, China; 2 Clinical Medical College of Henan University of Science and Technology, Luoyang, China; 3 Department of Neurology Ward 1, Pingdingshan First People’s Hospital, Pingdingshan, China; 4 Henan Vocational College of Massage, Luoyang, China

**Keywords:** cognitive impairment, motor progression, neuroimmune-enriched multibiofluid proteomic index, neuroimmunology, Parkinson’s disease, PD-MCI, PPMI (Parkinson’s Progression Markers Initiative), proteomics

## Abstract

Progression in early Parkinson's disease (PD) is heterogeneous, motivating transparent biological markers of group-level progression context. We analyzed longitudinal Parkinson’s Progression Markers Initiative (PPMI) data downloaded on 31 May 2026. The neuroimmune-enriched multibiofluid proteomic index (NEMPI) used 718 quality-control-eligible cerebrospinal fluid and plasma markers, organized into four source-context modules with equal marker and module weights. Direct normalized protein quantification (NPQ) z-standardization was used for the primary reporting implementation, with the log1p implementation examined as a preprocessing sensitivity analysis. Among the 521 participants in the same-sample Cox comparison (173 first observed cognitive-status abnormalities), adding NEMPI to demographic, clinical, alpha-synuclein seed amplification assay, dopamine-transporter imaging, and apolipoprotein E (APOE) epsilon4 covariates yielded an NEMPI hazard ratio (HR) of 1.287 per 1-SD higher value (95% confidence interval (CI): 1.102–1.502; P = 0.001) and improved likelihood-based fit, but the C-index increment was small (0.0032; bootstrap 95% CI: -0.0093 to 0.0239). The consecutive-abnormality association was stronger but had fewer events, with fewer than 10 events per parameter; the persistent/irreversible estimate was weaker, and its confidence interval crossed 1. Higher NEMPI was also associated with a modest group-average Montreal Cognitive Assessment (MoCA) and motor trajectory differences, with 5-year translations below published minimal clinically important difference (MCID) ranges. Results were similar under 5-year censoring, discrete-time analysis, stabilized inverse-probability weighting, age analyses, and measured confounder or preanalytic adjustments. NEMPI is therefore interpreted as an abundance-based, neuroimmune-enriched, multibiofluid proteomic composite associated with broad cognitive-motor progression, not as a patient-level prediction tool, clinical threshold, cognition-specific biomarker, or causal mechanism.

## Introduction

Parkinson’s disease (PD) progression is heterogeneous across patients, clinical domains, and biological contexts. Even among individuals enrolled early in the disease course, the pace and pattern of motor disability, cognitive impairment, autonomic involvement, and other non-motor milestones vary substantially. This heterogeneity is not only a statistical challenge; it reflects that PD is a multi-system disorder shaped by synuclein pathology, dopaminergic degeneration, genetic background, co-pathology, aging-related vulnerability, and immune or inflammatory processes. Large longitudinal cohorts such as the Parkinson’s Progression Markers Initiative (PPMI) were designed in part to address this problem by integrating clinical, imaging, biospecimen, and molecular data across repeated follow-up (1–4). Recent multi-cohort work further supports the concept that PD progression can be stratified into distinct longitudinal progression patterns, reinforcing the need for biologically anchored markers of broad progression patterns (5).

Cognitive and motor progression are two clinically central but biologically heterogeneous dimensions of early PD. Cognitive impairment can occur early, affects quality of life and functional independence, and may involve attentional-executive, visuospatial, memory, and mixed profiles. Motor progression also varies, and motor rating scales remain important longitudinal anchors despite the influence of symptomatic treatment and clinical fluctuation. Current evidence suggests that cognitive and motor trajectories may reflect overlapping but non-identical biological substrates, including frontostriatal dysfunction, posterior cortical involvement, cholinergic and dopaminergic pathway disruption, Lewy body burden, AD-type co-pathology, and systemic vulnerability (6–8).

Neuroimmune and proteomic processes may provide a complementary biomarker context for progression heterogeneity. Inflammatory and proteomic markers are promising for translation, but single-marker approaches can be noisy, tissue-dependent, and difficult to reproduce. An internally specified, interpretable index-level proteomic signature may provide a more stable group-level biological context than post-outcome individual-marker selection. Recent cerebrospinal fluid (CSF) and plasma proteomic studies in PD have shown that multi-protein signatures can capture diagnostic, progression-related, and biologically heterogeneous disease contexts, supporting the rationale for interpretable proteomic composite constructs (9–13). Neuroimmune reviews and inflammatory biomarker syntheses further support the relevance of microglial, astroglial, cytokine, and chemokine biology in PD and related neurodegenerative disorders (14–17), while recent PPMI multibiofluid proteomic work supports the feasibility of CSF/plasma proteomic biomarker discovery in early PD (18).

Existing biomarker approaches in PD have made major advances but do not fully resolve progression heterogeneity. Alpha-synuclein seed amplification assay (SAA) provides important synuclein-pathology context and has reshaped biological classification of PD cohorts. Dopamine transporter single-photon emission computed tomography (DAT-SPECT) reflects presynaptic dopaminergic integrity and remains a key imaging context. CSF Alzheimer disease (AD) biomarkers, including amyloid-beta and tau measures, provide co-pathology context relevant to cognition in PD. However, these canonical markers do not exhaust the biological space of cognitive and motor progression, particularly as the field moves toward biomarker-informed staging, monitoring, and targeted-intervention frameworks (19, 20). Similarly, prior inflammatory biomarker studies often focus on individual cytokines or single glial markers, which can be biologically informative but vulnerable to assay noise, pathway redundancy, and outcome-driven selection (16, 21–24).

A key translational question is whether a single-score, module-structured composite index provides information on group-level longitudinal progression after adjustment for canonical PD biomarker contexts such as SAA, DAT-SPECT, CSF AD biomarkers, and genetic background, without being framed as a patient-level decision rule (9, 10, 19, 20).

In this study, we evaluated an abundance-based, neuroimmune-enriched multibiofluid proteomic index, termed NEMPI, in PPMI participants with PD who were cognitively normal at baseline. We examined the first observed cognitive-status abnormality as the primary outcome, cognitive-state stability and longitudinal cognitive-motor trajectories as secondary outcomes, and incremental model fit without construing NEMPI as a patient-level prediction instrument.

## Materials and methods

### Study design and data source

This was an observational longitudinal cohort analysis using data from PPMI. PPMI is an international, multicenter, longitudinal study that collects standardized clinical, imaging, biospecimen, genetic, and biomarker data from participants with PD, prodromal features, genetic risk, and healthy controls (1, 2). For this analysis, PPMI data were downloaded on 31 May 2026.

The analysis retained the outcome-free marker-eligibility process, the fixed 718-marker whitelist, four source-context modules, technical abundance directions, equal-weighting rules, and the primary outcome and model hierarchy. No marker, module, weight, direction, covariate set, threshold, or outcome definition was altered on the basis of outcome associations, P values, or effect estimates.

### Participants

Eligible participants were PPMI baseline PD participants who had baseline cognitive status without mild cognitive impairment (MCI) or dementia, baseline proteomic availability sufficient for NEMPI construction, and follow-up information for the relevant longitudinal outcomes. Baseline PD status followed PPMI cohort definitions and standardized diagnostic procedures aligned with movement-disorder research criteria (1, 2, 25, 26). Baseline cognitive eligibility required the absence of MCI or dementia at the baseline visit, using PPMI cognitive categorization fields and longitudinal cognitive follow-up.

Participants were excluded from a given analysis if the required NEMPI, outcome follow-up, or prespecified model covariates were unavailable. The candidate analysis set contained 561 participants; 522 contributed to the descriptive baseline set; and 521 participants with 173 events contributed to the same-sample primary Cox comparison. Denominators for secondary analyses were reported separately.

### Neuroimmune proteomic data and quality control

Baseline neuroimmune-enriched proteomic data were derived from four Nucleic Acid-Linked Immuno-Sandwich Assay (NuLISA) (27) panels: the CSF CNS disease panel, CSF inflammation panel, plasma CNS disease panel, and plasma inflammation panel. Platform-provided normalized protein quantification (NPQ) values were used as the marker input. Protein-marker eligibility was evaluated before outcome modeling. Quality control (QC) considered marker availability, missingness, duplicate or ambiguous rows, assay interpretability, panel and biofluid source, batch or plate metadata where available, and assay-level suitability.

The pre-outcome marker-eligibility algorithm applied sequential support-only, numeric-availability, missingness, observation-count, and duplicate-ambiguity rules. Among the 11,681 panel-by-biofluid marker rows, 53 prespecified support-only rows were set aside, 4 rows were unusable because no numeric baseline values were available, and 10,906 rows had missingness greater than 0.80 using 561 candidates with non-missing baseline visit order as the denominator; 718 rows were eligible for the primary index. The additional missingness branch greater than 0.40, the fewer-than-30-observations branch, and the duplicate-ambiguity branch caused no further exclusions. Duplicate participant-marker rows were collapsed by the median; all 357 duplicate groups had two values, so the median equaled the arithmetic mean. Numeric conversion failures became missing. No project-level limit of detection (LOD), lower limit of quantification (LLOQ), or upper limit of quantification (ULOQ) substitution was applied; assay WARN flags were retained and described, and Tukey 1.5×IQR outliers were counted but neither deleted nor winsorized. No outcome, P value, or effect estimate entered eligibility, direction, weighting, or module assignment.

### Construction of the neuroimmune-enriched multibiofluid proteomic index

For the primary reporting implementation, each eligible marker j was directly standardized among candidate participants with an observed value as z_ij = (NPQ_ij-mean_j)/SD_j, where SD_j was the sample standard deviation calculated using an n−1 denominator. The log1p marker-z implementation was examined as a preprocessing sensitivity analysis: nonnegative NPQ values were transformed as ln(1+NPQ) before within-marker z-standardization; zero mapped to zero, missing values remained missing, and a marker with any negative observed value would have retained the untransformed NPQ before standardization. All 718 eligible markers were nonnegative. Both implementations used the identical 718-marker whitelist, four modules, technical directions, and equal-weighting rules.

The four modules contained 114 CSF CNS-disease, 243 CSF-inflammation, 116 plasma CNS-disease, and 245 plasma-inflammation markers. Within each module, the participant score was the arithmetic mean of available marker z scores; the raw NEMPI was the arithmetic mean of available module scores, and the reported direct-z NEMPI was standardized across the 557 score-available participants. Thus, markers were equally weighted within modules, and modules were equally weighted across the index. There was no minimum marker or module coverage threshold: 551 participants had all four modules, 6 had two modules, and 4 had no score. Technical direction retained higher abundance as measured and was never flipped using outcomes; it should not be interpreted as evidence that every included protein is biologically harmful.

The fixed support-only boundary included NfL/NEFL, GFAP, sTREM2, YKL-40, IL-6, S100B, NRGN, and CSF total tau contexts; these markers did not enter the primary NEMPI. Support-only and individual-marker analyses remained biological-context assessments and were not used to alter the composite.

### Biological annotation of NEMPI

Biological annotations were applied for interpretation only and did not change the 718-marker whitelist, technical direction, module assignment, or weight. Broad proteomic context accounted for 320 markers (44.6%); adaptive immune/cytokine/chemokine, CNS-enriched, tissue-remodeling/vascular/stress, microglial/innate immune, neuroaxonal/synaptic injury-support, and astroglial/glial-stress categories accounted for 264, 58, 36, 22, 12, and 6 markers, respectively. Immune, glial, and vascular/stress categories together comprised 328 of 718 markers (45.7%); NEMPI is therefore described as neuroimmune-enriched rather than purely neuroimmune (**Supplementary Table S16**).

Annotations covered adaptive immune/cytokine/chemokine, microglial/innate immune, astroglial/glial-stress, tissue-remodeling/vascular-stress, broad CNS/proteomic, and injury-support contexts. Complete marker-level eligibility, source, annotation, role, and direction fields are provided in **Supplementary Data 1**.

### Outcomes

The primary outcome was first observed cognitive-status abnormality: the first follow-up visit with PPMI COGSTATE = 2 or 3 among participants with baseline COGSTATE = 1, timed at that visit. Consecutive abnormality required COGSTATE = 2 or 3 at two consecutive observed visits and was timed at the first visit in the qualifying sequence. Persistent/irreversible abnormality required that all observed visits after the first abnormal visit remained abnormal and was timed at the first abnormal visit. Recovery was any subsequent COGSTATE = 1 after the primary event, and recurrent abnormality was a later COGSTATE = 2 or 3 after recovery. First PD-MCI, first dementia, and PD-MCI-to-dementia transitions were also enumerated. Participants without a qualifying event were censored at their last observed cognitive follow-up.

Secondary cognitive outcomes were longitudinal MoCA and two MoCA-derived domain candidate outcomes: an executive-attention item-sum candidate and a delayed-recall memory item-sum candidate. These MoCA-derived domain candidates were analyzed as supportive trajectory outcomes and should not be interpreted as independent multi-test neuropsychological composites. Details on item composition, scoring direction, and overlap considerations are provided in the **Supplementary Methods** (28). Secondary motor-context outcomes were Movement Disorder Society-Unified Parkinson’s Disease Rating Scale (MDS-UPDRS) II and MDS-UPDRS III, reflecting motor experiences of daily living and motor examination, respectively (6). These motor analyses were interpreted as progression-context analyses rather than as core clinical endpoints or decision instruments.

### Covariates

Primary covariates included demographic and baseline clinical variables selected before outcome modeling: age, sex, education, disease duration, and baseline MoCA for analyses of first observed cognitive-status abnormality. Covariates were selected *a priori* to balance demographic and clinical confounding control, canonical biomarker context, and events-per-parameter stability. Biological context covariates included SAA positivity, DAT-SPECT mean striatal-specific binding ratio (SBR), and apolipoprotein E (APOE) epsilon4 carrier status where available. SAA positivity was coded from Amprion alpha-synuclein seed amplification assay status as a binary positive flag; SAA-positive subset analyses were conducted as sensitivity analyses because SAA positivity was common in the cohort. DAT-SPECT was summarized using mean striatal SBR, with caudate, putamen, lowest-striatal, and mean-striatal values retained for context.

SAA, DAT-SPECT, CSF AD biomarkers, and genetic variables were treated as canonical biological context. They were not considered replacements for NEMPI and were not used to construct NEMPI (21–24).

### Statistical analysis

Baseline characteristics were summarized overall and descriptively by NEMPI tertile. Tertiles were used only for tables and curves and did not define a clinical threshold. The primary exposure was continuous NEMPI per 1 SD higher value.

The primary same-sample comparison used Cox regression among 521 participants with 173 first observed cognitive-status abnormalities. The Base model contained age, sex, education, PD duration, baseline MoCA, alpha-synuclein seed amplification assay status, DAT mean striatal-SBR, and APOE epsilon4 carriage; the Extended model added direct-z NEMPI. We reported the NEMPI hazard ratio (HR), likelihood-ratio test (LRT), Akaike information criterion (AIC), Bayesian information criterion (BIC), Harrell C-index, and the C-index increment with a 1,000-replicate participant-level bootstrap confidence interval. Proportional-hazards diagnostics used Schoenfeld residuals.

Secondary analyses included Cox models for consecutive and persistent/irreversible abnormality; a participant-clustered complementary log–log person–period model with log interval length as an offset; full-follow-up and 5-year administratively censored Cox models; age-NEMPI correlations, an age-by-NEMPI interaction, age-residualized NEMPI, and fixed age strata (<60, 60–69, and ≥70 years) retaining continuous age. Longitudinal MoCA and MDS-UPDRS II/III models included NEMPI-by-time interactions. Linear time, time restricted to 5 years, natural cubic spline time with 3 degrees of freedom, and a fixed 5-year piecewise-linear form were evaluated under the prespecified model sequence. Random intercepts and time slopes were preferred, with simplification only when required for convergence.

### Sensitivity analyses and diagnostics

Selection analyses compared the 521 included and 40 excluded participants within the 561-participant candidate analysis set using absolute standardized mean differences across demographic, disease-duration, cognitive-follow-up, visit-order, and biomarker/index-availability variables. Stabilized inverse-probability weights for inclusion were truncated at the first and 99th percentiles; post-weighting balance was assessed between the weighted included sample and the full candidate set, and positivity, effective sample size, and robust-standard-error weighted Cox results were retained with computational warnings. Multiple imputation was to begin only if ordinary covariate missingness removed more than 5% of the otherwise eligible sample and was not triggered. Death dates were adjudicated from prespecified sources; undated deaths were not imputed, and last follow-up was not substituted. Cause-specific Cox and Fine-Gray analyses were restricted to reliable-date participants.

Motor-context analyses separated explicit OFF and ON MDS-UPDRS III records, collapsed same-visit same-state duplicates by the median, and never averaged ON and OFF records. Secondary medication-adjusted models used time-varying levodopa equivalent daily dose (LEDD), treatment status, and medication state. MDS-UPDRS II sensitivities added baseline or time-varying LEDD over full and 5-year follow-up. Additional prespecified one-at-a-time Cox sensitivities included BMI, comorbidity burden and categories, active anti-inflammatory medication, or noncollinear preanalytic variables. Renal function, total protein, and sample loading were not available, and no proxy variables were constructed. MoCA executive-attention and memory measures were treated only as MoCA-derived item-sum candidates, not validated domain composites.

The primary inferential anchor and covariate hierarchy remained unchanged. Cognitive-state alternatives, longitudinal cognitive and motor outcomes, age strata, weighting, competing risk, technical context, extended confounding, and preprocessing analyses were secondary, sensitivity, exploratory, or diagnostic as designated. Negative, attenuated, nonconvergent, low-events-per-parameter, and unavailable-data findings were retained.

## Results

### Cohort construction and baseline characteristics

**Figure 1** summarizes cohort construction, outcome-free marker eligibility, the fixed four-module architecture, primary and secondary analysis contexts, and the interpretation boundary. The candidate analysis set contained 561 participants; 522 were included in descriptive summaries, and 521 with 173 events entered the primary Cox analysis.

**Figure 1:**
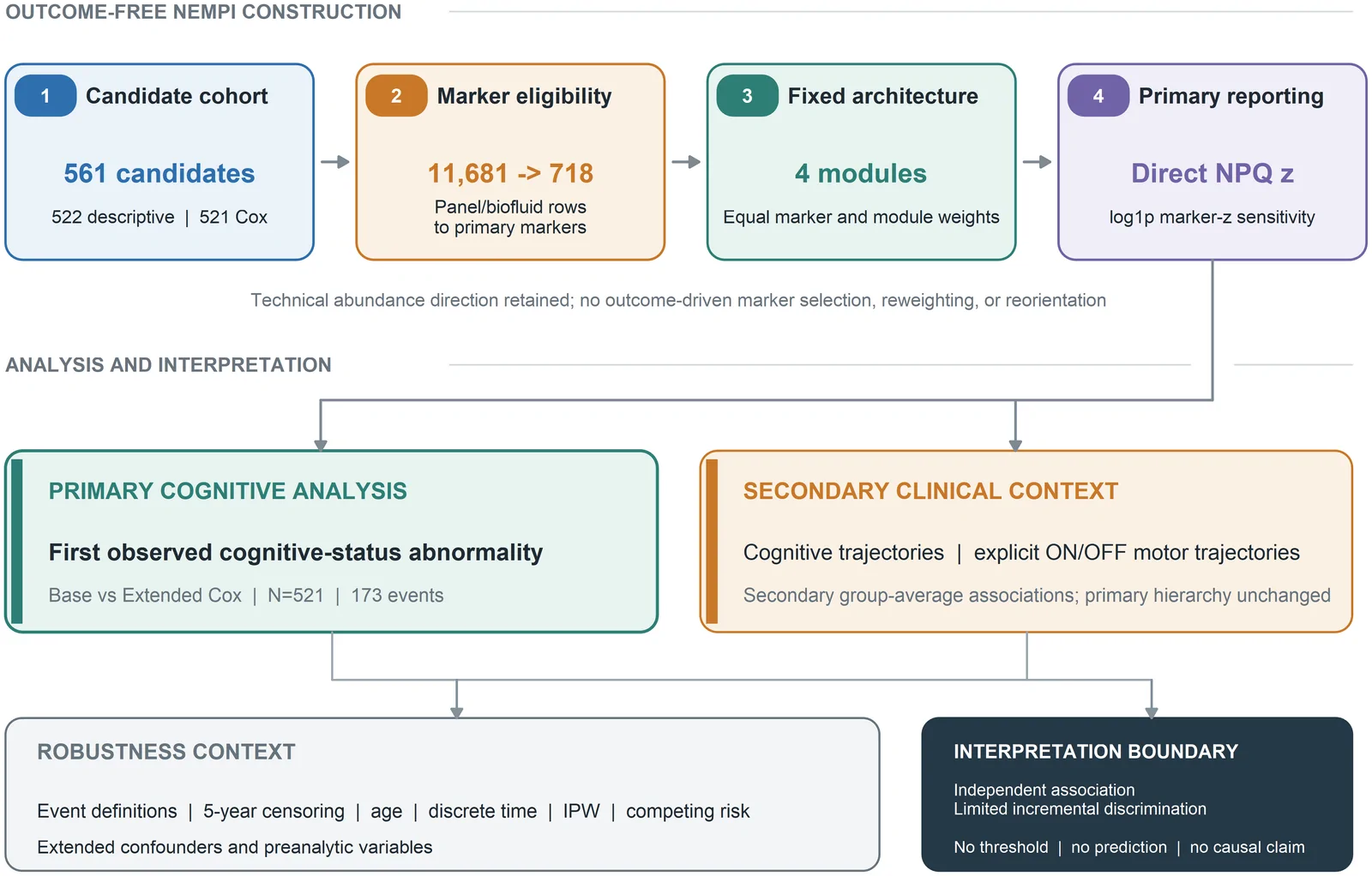
Study design and analysis flow. The candidate cohort, pre-outcome marker-eligibility flow, fixed four-module architecture, primary reporting implementation, first observed cognitive-status abnormality, secondary cognitive-motor context, robustness analyses, and interpretation boundary are shown. Direct NPQ z-standardization was used for primary reporting; log1p marker-z was a preprocessing sensitivity. NEMPI was analyzed continuously; no patient-level threshold was defined. CNS, central nervous system; Cox, Cox proportional hazards regression; CSF, cerebrospinal fluid; MCI, mild cognitive impairment; NPQ, normalized protein quantification; NEMPI, neuroimmune-enriched multibiofluid proteomic index; PD, Parkinson’s disease; and PPMI, Parkinson’s Progression Markers Initiative.

Baseline characteristics are shown in **Table 1**. The descriptive set had a mean age of 62.5 years, 42.0% female participants, mean education of 16.1 years, median PD duration of 0.81 year, and a mean baseline MoCA of 27.0. The higher descriptive NEMPI tertile was older. In the primary-analysis clinical context, median follow-up was 4.00 years, the median number of cognitive assessments was 4, mean baseline MDS-UPDRS II was 6.75, baseline LEDD had a median of 0, and 26.5% were receiving PD treatment. BMI, comorbidity burden, anti-inflammatory medication, and explicit ON/OFF record availability are also reported.

**Table 1:** Baseline characteristics by descriptive NEMPI tertile, with extended clinical context.

Characteristic	N available	Overall	Lower	Middle	Higher
Descriptive N	522	522	174	174	174
Age, years	522	62.5 (9.7)	58.4 (9.6)	61.7 (9.3)	67.5 (8.0)
Female sex, n (%)	522	219 (42.0)	77 (44.3)	72 (41.4)	70 (40.2)
Education, years	522	16.1 (3.1)	16.1 (2.8)	16.2 (3.2)	15.9 (3.2)
PD duration, years	522	0.81 (0.42–1.86)	0.84 (0.43–1.85)	0.77 (0.40–1.73)	0.80 (0.43–1.96)
Baseline MoCA	521	27.0 (2.5)	27.2 (2.4)	26.8 (2.5)	26.8 (2.4)
First observed cognitive-status abnormality, n (%)	522	173 (33.1)	47 (27.0)	46 (26.4)	80 (46.0)
SAA positive, n (%)	522	472 (90.4)	163 (93.7)	158 (90.8)	151 (86.8)
DAT mean striatal SBR	522	1.4 (0.4)	1.4 (0.4)	1.4 (0.4)	1.4 (0.4)
APOE epsilon4 carrier, n (%)	522	125 (23.9)	38 (21.8)	50 (28.7)	37 (21.3)
Follow-up duration, years	521	4.00 (2.17–7.92)	3.92 (2.50–8.50)	3.21 (2.17–7.77)	4.08 (2.11–7.06)
Cognitive assessments, count	521	4 (3–7)	5 (3–8)	4 (3–7)	4 (3–7)
Baseline MDS-UPDRS II	519	6.75 (4.09)	6.73 (3.97)	6.47 (3.46)	7.06 (4.73)
Baseline MDS-UPDRS III, explicit OFF	116	24.27 (11.37)	24.38 (10.91)	21.03 (11.24)	26.52 (11.57)
Baseline MDS-UPDRS III, explicit ON	129	17.74 (9.47)	17.47 (9.46)	17.03 (10.38)	18.57 (8.86)
Baseline LEDD	521	0 (0–220)	0 (0–200)	0 (0–155)	0 (0–300)
PD treatment, n (%)	521	138 (26.5)	47 (27.2)	41 (23.6)	50 (28.7)
BMI, kg/m^2^	518	26.46 (4.80)	25.96 (5.33)	26.59 (4.57)	26.83 (4.46)
Active comorbidity burden	521	0 (0–3)	0 (0–2)	0 (0–2)	0 (0–5)
Active anti-inflammatory medication, n (%)	521	136 (26.1)	35 (20.2)	41 (23.6)	60 (34.5)
Any explicit ON record, n (%)	521	482 (92.5)	164 (94.8)	159 (91.4)	159 (91.4)
Any explicit OFF record, n (%)	521	433 (83.1)	147 (85.0)	141 (81.0)	145 (83.3)

Values are mean (SD), median (IQR), or n (%). Direct-z NEMPI tertiles are descriptive only and do not define clinical thresholds; denominators vary according to variable availability, and no between-tertile P-value screening was performed. Abbreviations: APOE, apolipoprotein E; BMI, body mass index; DAT, dopamine transporter; IQR, interquartile range; LEDD, levodopa equivalent daily dose; MDS-UPDRS, Movement Disorder Society-Unified Parkinson’s Disease Rating Scale; MoCA, Montreal Cognitive Assessment; NEMPI, neuroimmune-enriched multibiofluid proteomic index; OFF/ON, medication examination state; PD, Parkinson’s disease; SAA, alpha-synuclein seed amplification assay; SBR, specific binding ratio; and SD, standard deviation.

### NEMPI construction and biological annotation

The marker-eligibility flow went from 11,681 rows to 718 primary markers after 10,906 high-missingness, 53 support-only, and 4 unusable rows were excluded; no row was additionally excluded by the 0.40 missingness, fewer-than-30, or duplicate-ambiguity branches. The fixed four modules and all equal weights and technical abundance directions were retained. The resulting index is described as neuroimmune-enriched because it includes both immune-related and broader CNS/proteomic contexts.

### NEMPI and first observed cognitive-status abnormality

In the same 521 participants and 173 events, the Base model had AIC 1797.93, BIC 1831.98, and C-index 0.6731. The Extended model had AIC 1789.80, BIC 1828.10, and C-index 0.6764; the likelihood-ratio statistic was 10.13 (P = 0.0015). NEMPI was associated with the first observed cognitive-status abnormality (HR per 1 SD = 1.287, 95% CI: 1.102–1.502; P = 0.001). The C-index increment was only 0.0032, and its 1,000-bootstrap 95% CI crossed 0 (-0.0093 to 0.0239). Thus, the result supports independent association and better likelihood-based fit, but not improved discrimination. The log1p preprocessing sensitivity was similar (HR = 1.306, 95% CI: 1.112–1.532; P = 0.001), and direct-z and log1p scores were highly concordant (Pearson r = 0.9964; Spearman rho = 0.9981).

The consecutive-abnormality analysis included 77 events and yielded HR 1.525 (95% CI: 1.205 –1.931; P < 0.001), with 8.6 events per parameter. The persistent/irreversible analysis included 115 events and yielded HR 1.182 (95% CI: 0.977–1.431; P = 0.086); its confidence interval crossed 1. First PD-MCI occurred in 171 participants, first dementia in 23, recovery after first abnormality in 103, recurrent abnormality after recovery in 56, and PD-MCI-to-dementia progression in 19. Median times and the complete transition summary are reported in **Supplementary Table S3**.

The complementary log-log interval model gave HR 1.304 (95% CI: 1.109–1.534; P = 0.001). Five-year administrative censoring retained 122 events and gave HR 1.274 (95% CI: 1.051–1.543; P = 0.013). NEMPI correlated with age (Pearson r = 0.384; Spearman rho = 0.399), but the age-by-NEMPI interaction was null (HR 0.999, 95% CI: 0.983–1.016; P = 0.924), and age-residualized results remained supportive. Fixed age strata were imprecise because each had fewer than 10 events per parameter and are not interpreted as differential subgroup effects.

Within the 561-candidate set, the largest included-versus-excluded imbalances were direct-z NEMPI availability (100.0% vs 90.0%; absolute SMD = 0.471) and the mean number of cognitive follow-up visits (6.96 vs 5.53; absolute SMD = 0.406). Smaller imbalances were observed for education (16.05 vs 15.23 years; absolute SMD = 0.249), SAA availability (100.0% vs 97.5%; absolute SMD = 0.226), and female sex (41.8% vs 50.0%; absolute SMD = 0.164). Age and PD duration were closely balanced (absolute SMDs 0.010 and 0.019, respectively), and DAT availability showed no imbalance among evaluable candidates. After weighting, the maximum absolute SMD between the weighted included sample and the full candidate set was 0.042. Stabilized inclusion weights were narrow (median 0.992; range after truncation 0.941-1.125), the effective sample size was 520.1, and no clear positivity violation was identified. The weighted Cox estimate was similar (HR 1.287, 95% CI: 1.097–1.508; P = 0.002), but an optimizer precision-loss warning persisted; this analysis does not establish removal of selection bias. The primary association and selected sensitivity analyses are summarized in **Figure 2**. The earlier source cohort could be reconstructed only as aggregate flow (1,159 to 575 to 561), not for individual-level comparison. Ordinary covariate missingness removed 1 of 522 participants (0.19%), so the prespecified > 5% multiple-imputation trigger was not reached.

**Figure 2:**
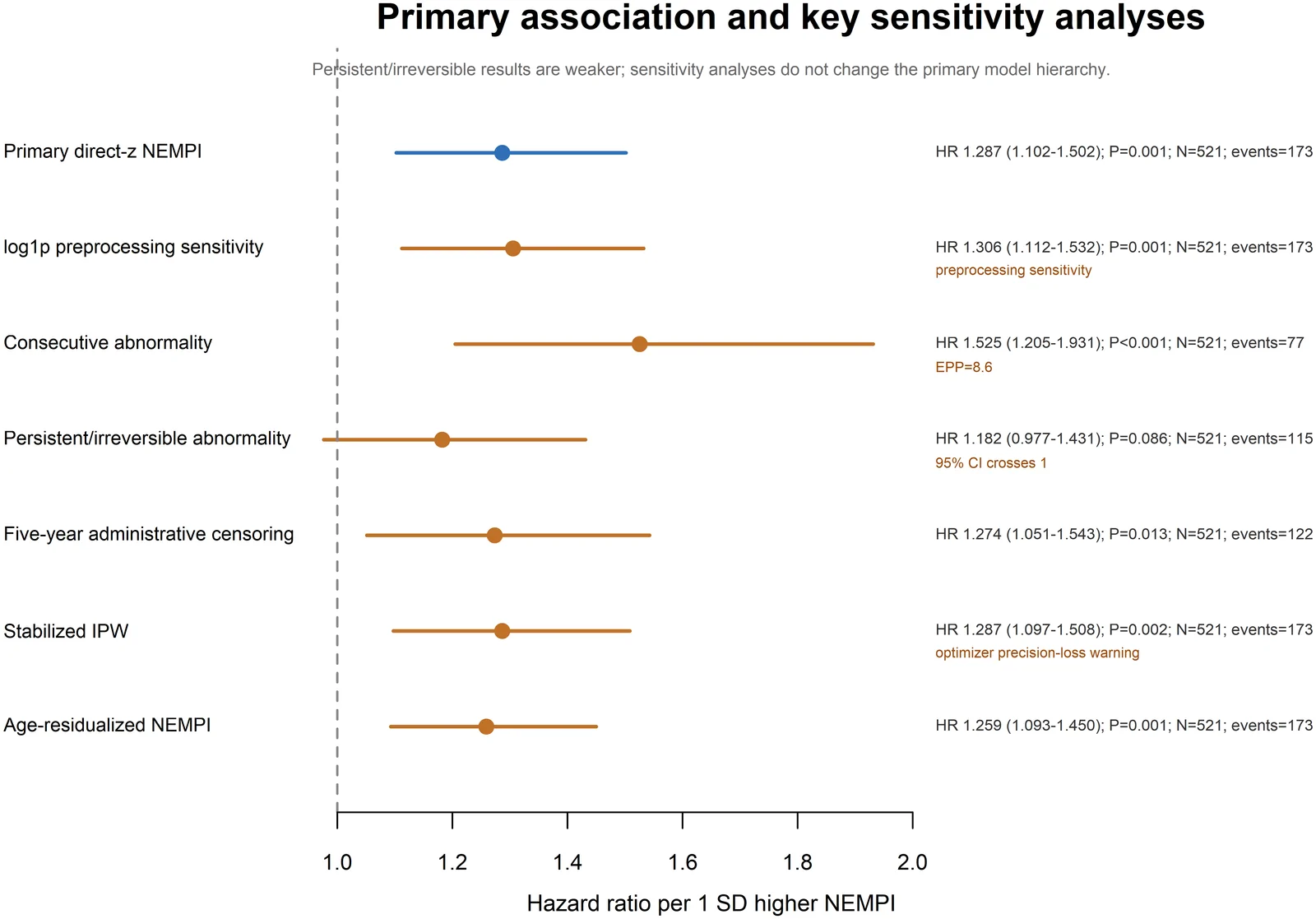
Primary association and selected sensitivity analyses. Hazard ratios are per 1 SD higher NEMPI; points indicate estimates and lines indicate 95% confidence intervals. The persistent/irreversible estimate crosses 1, consecutive abnormality has events per parameter below 10, and the IPW estimate retains an optimizer precision-loss warning. Sensitivity analyses do not change the primary model hierarchy. CI, confidence interval; EPP, events per parameter; HR, hazard ratio; IPW, inverse-probability weighting; NEMPI, neuroimmune-enriched multibiofluid proteomic index; and SD, standard deviation.

Among the 519 participants with reliable death dates, the cause-specific estimate was HR 1.317 (95% CI: 1.125–1.542; P < 0.001). No dated death occurred before a cognitive event or censoring within the risk window; Fine-Gray therefore produced the same point estimate and added no independent information about competing events. Two death-positive participants lacked dates and were excluded without imputation. Descriptive and covariate-standardized tertile curves are presented only as visualization, not as clinical thresholds (**Supplementary Figure S3**).

### Source-context decomposition and NEFL-adjusted sensitivity

In secondary source-context decomposition models, the four NEMPI source-context module scores were evaluated separately without altering the primary NEMPI definition or model hierarchy. In Cox models for first observed cognitive-status abnormality, the plasma CNS disease module showed the clearest supportive association (HR 1.26, 95% CI: 1.08 to 1.47, P = 0.003, BH-FDR q = 0.013), followed by plasma inflammation (HR 1.25, 95% CI: 1.06 to 1.47, P = 0.008, q = 0.015) and CSF inflammation (HR 1.28, 95% CI: 1.02 to 1.61, P = 0.037, q = 0.049). These estimates are interpreted as exploratory decomposition rather than evidence that any single module drives the primary NEMPI association.

In CSF/plasma decomposition, the mean plasma module showed a stronger supportive Cox estimate than the mean CSF module. In the mutually adjusted model, the plasma module mean remained directionally supportive (HR 1.26, 95% CI: 1.08 to 1.47, P = 0.004), with low collinearity between CSF and plasma module means (VIF 1.01). This supports a cautious source-context interpretation in which systemic immune/proteomic context may contribute to the broad progression-associated signal.

NEFL-adjusted sensitivity analyses used sample-matched Cox models with support-only NuLISA NEFL measures. NEFL adjustment did not attenuate the NEMPI association (CSF NuLISA NEFL-adjusted model: HR 1.40, 95% CI: 1.16 to 1.69, P < 0.001; plasma NuLISA NEFL-adjusted model: HR 1.32, 95% CI: 1.12 to 1.56, P < 0.001). These findings are best described as an injury-support overlap assessment; they do not establish independence from neuronal injury or a separate biological pathway.

### NEMPI and longitudinal cognitive trajectories

Higher NEMPI was associated with a faster full-follow-up MoCA trajectory (N = 517; 2,520 observations; beta per 1 SD NEMPI per year = -0.060, 95% CI: -0.103 to -0.017; P = 0.006). The 5-year restricted linear estimate was also negative (beta = -0.089, 95% CI: -0.151 to -0.026; P = 0.006), and spline and fixed 5-year piecewise forms retained joint evidence of time-varying association. The full-model 5-year translation was -0.299 MoCA points, or 11.4% of the baseline SD.

MoCA executive-attention and memory variables are reported only as MoCA-derived item-sum candidates. Their item composition, complete-item missingness rules, and possible measurement overlap with COGSTATE are described in **Supplementary Methods**. They are not validated domain composites and do not support an executive-function-specific claim.

### NEMPI and motor progression

Full-follow-up MDS-UPDRS II worsened by 0.162 points per year per 1 SD higher NEMPI (95% CI: 0.062–0.262; P = 0.001). Baseline-LEDD and time-varying-LEDD full-follow-up estimates remained positive, but both corresponding 5-year estimates were nonsignificant (P = 0.120 and P = 0.149). Explicit OFF-state MDS-UPDRS III had beta = 0.302 (95% CI: 0.014–0.590; P = 0.040; N = 433; 1,951 observations), and explicit ON-state MDS-UPDRS III had beta = 0.313 (95% CI: 0.125–0.502; P = 0.001; N = 482; 2,928 observations). ON-state and combined medication-adjusted models converged, whereas all prespecified OFF-state medication-adjusted attempts were singular/nonconvergent, and no reliable replacement estimate was reported.

Group-average translations per 1 SD higher NEMPI were -0.180 and -0.299 MoCA points, 0.487 and 0.812 MDS-UPDRS II points, and 0.905 and 1.509 explicit OFF-state MDS-UPDRS III points at 3 and 5 years, respectively. The 5-year differences were 11.4%, 18.6%, and 13.3% of their baseline SDs and remained below published minimal clinically important difference (MCID) ranges (29–31). The selected full-follow-up cognitive and explicit medication-state motor trajectory results are summarized in **Figure 3** and **Table 3**.

**Figure 3:**
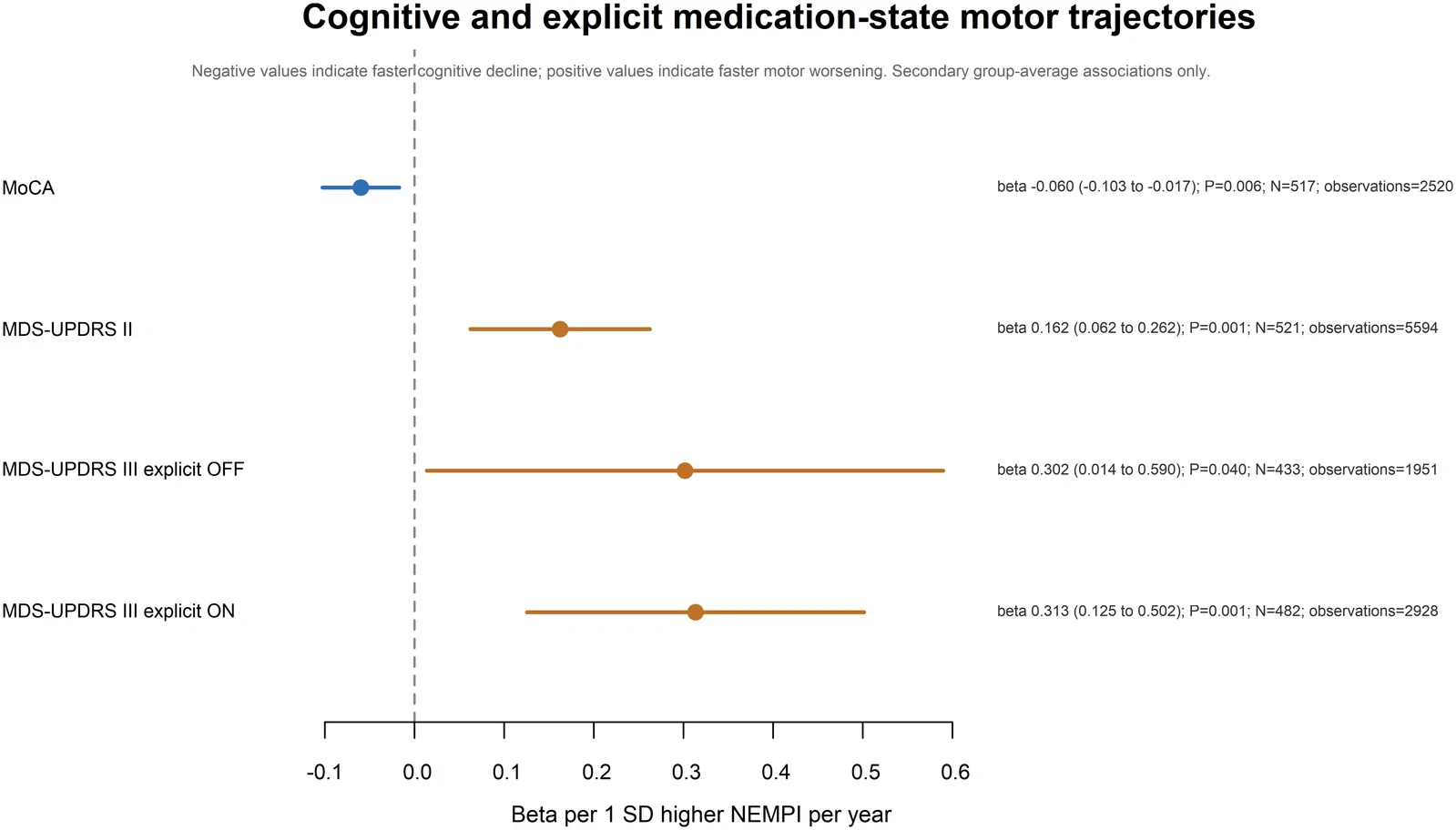
Cognitive and explicit medication-state motor trajectories. Coefficients are NEMPI-by-time interactions per 1 SD higher NEMPI per year. Negative values indicate faster MoCA decline; positive values indicate faster MDS-UPDRS worsening. Estimates are secondary group-average associations. CI, confidence interval; MDS-UPDRS, Movement Disorder Society-Unified Parkinson’s Disease Rating Scale; MoCA, Montreal Cognitive Assessment; NEMPI, neuroimmune-enriched multibiofluid proteomic index; and SD, standard deviation.

### Extended confounder and preanalytic sensitivity analyses

One-at-a-time sensitivities adding BMI (N = 518; HR 1.288), comorbidity burden and prespecified categories (N = 521; HR 1.250), active anti-inflammatory medication (N = 521; HR 1.276), or noncollinear preanalytic variables (N = 515; HR 1.287) were similar in direction and magnitude to the primary estimate; VIF values were below 2.1. They reduce concern about measured confounding but do not establish causal independence. Smoking was descriptive/conditional only. Renal function, total protein, and sample loading were unavailable, and no substitutes were constructed.

## Discussion

The abundance-based NEMPI was associated with the first observed cognitive-status abnormality, faster cognitive decline, and faster motor progression in baseline non-MCI PD participants from PPMI. The combined pattern supports NEMPI as a group-level marker associated with broad cognitive-motor progression in a human longitudinal cohort, aligning with recent work emphasizing longitudinal progression heterogeneity and the need for biologically informative progression markers in PD (5, 19, 20). The findings should not be interpreted as cognition-specific or as a patient-level decision instrument.

The same-sample comparison clarifies the incremental result (**Table 2**). Adding NEMPI improved likelihood-based fit and reduced AIC and BIC, but increased the C-index by only 0.0032, with a bootstrap confidence interval crossing 0. The appropriate interpretation is an independent association with limited incremental discrimination, not prediction improvement, clinical utility, or a decision threshold.

**Table 2:** The same-sample Base versus Extended Cox models for the first observed cognitive-status abnormality.

Model	N	Events	NEMPI HR (95% CI)	P value	LRT	AIC	BIC	C-index/change
Base	521	173	Not applicable	Not applicable	Reference	1797.93	1831.98	0.6731
Extended: Base + NEMPI	521	173	1.287 (1.102–1.502)	0.001	10.13; P = 0.0015	1789.80	1828.10	0.6764
Extended minus Base	521	173	Not applicable	Not applicable	1 df	-8.13	-3.88	0.0032 (-0.0093 to 0.0239)

Base covariates were age, sex, education, PD duration, baseline MoCA, SAA status, DAT mean striatal SBR, and APOE epsilon4 carriage. The C-index change confidence interval used 1,000 participant-level bootstrap replicates and crossed 0. Abbreviations: AIC, Akaike information criterion; APOE, apolipoprotein E; BIC, Bayesian information criterion; CI, confidence interval; DAT, dopamine transporter; HR, hazard ratio; LRT, likelihood-ratio test; MoCA, Montreal Cognitive Assessment; NEMPI, neuroimmune-enriched multibiofluid proteomic index; PD, Parkinson’s disease; SAA, alpha-synuclein seed amplification assay; and SBR, specific binding ratio.

**Table 3:** Selected full-follow-up cognitive and explicit medication-state motor trajectories.

Outcome	N	Observations	Beta/year (95% CI)	P value	3-year difference	5-year difference	5-year % baseline SD
MoCA	517	2,520	-0.060 (-0.103 to -0.017)	0.006	-0.180	-0.299	-11.4%
MDS-UPDRS II	521	5,594	0.162 (0.062–0.262)	0.001	0.487	0.812	18.6%
MDS-UPDRS III explicit OFF	433	1,951	0.302 (0.014–0.590)	0.040	0.905	1.509	13.3%
MDS-UPDRS III explicit ON	482	2,928	0.313 (0.125–0.502)	0.001	0.940	1.567	Not evaluated

Beta is the NEMPI-by-time coefficient per 1 SD higher NEMPI per year. Three- and five-year values are group-average linear translations. The 5-year MoCA, MDS-UPDRS II, and explicit OFF-state MDS-UPDRS III differences were below published MCID ranges and are not clinical thresholds. Abbreviations: CI, confidence interval; MCID, minimal clinically important difference; MDS-UPDRS, Movement Disorder Society-Unified Parkinson’s Disease Rating Scale; MoCA, Montreal Cognitive Assessment; NEMPI, neuroimmune-enriched multibiofluid proteomic index; OFF/ON, medication examination state; and SD, standard deviation.

The source-context and leave-one-module-out analyses provided supportive biological interpretation. The CSF CNS disease module alone had HR 1.10 (95% CI: 0.95–1.28; P = 0.218). Removing this module produced the largest leave-one-module-out estimate (HR 1.37, 95% CI: 1.15–1.63; P < 0.001), suggesting that the equal-weight structure may modestly dilute signals from the other modules. The CSF CNS disease module was nevertheless retained to preserve the fixed, outcome-independent architecture; it was neither deleted nor reweighted. The remaining source-context and leave-one-module-out results were directionally consistent but do not identify a causal driver or redefine the primary NEMPI (**Supplementary Table S17**). This interpretation is consistent with prior CSF proteomic and proteogenomic work showing that multi-protein profiles can capture PD-related biological heterogeneity and progression-relevant signatures (9, 10).

The index-level design remained transparent: a fixed 718-marker whitelist, four source-context modules, equal marker and module weights, and technical abundance directions were preserved without outcome-driven selection or reorientation. Direct NPQ z-standardization was used for the primary reporting implementation, with the log1p implementation examined as a preprocessing sensitivity analysis. Their near-identical ranking and association estimates indicate robustness to preprocessing without redefining the model hierarchy.

The cognitive findings are clinically coherent. First observed cognitive-status abnormality, defined as the first follow-up COGSTATE value of 2 or 3, was the primary outcome, and its association with MoCA decline provides longitudinal support for the event-based result. The exploratory MoCA-derived item-sum findings were directionally consistent, but these measures are not validated domain composites, may overlap with cognitive-status ascertainment, and do not support an executive-function-specific interpretation (7, 8). The memory association was smaller and should not be overemphasized. Taken together, the cognitive results support a longitudinal cognitive-decline association while avoiding the conclusion that NEMPI is specific to a single cognitive domain or to cognition alone.

The motor findings place NEMPI in a broader cognitive-motor progression context rather than establishing cognitive specificity. Explicit ON and OFF analyses avoided averaging medication states, and the converged ON and combined adjusted models were directionally similar. However, the OFF medication-adjusted model was singular, both 5-year MDS-UPDRS II LEDD sensitivities were nonsignificant, and the 5-year MoCA, MDS-UPDRS II, and OFF-state MDS-UPDRS III translations were below published MCID ranges. Clinical meaning is therefore modest and at the group level. Because the cohort did not include standardized short-interval replicate assessments designed to quantify test-retest error, cohort-specific measurement-variability bounds could not be estimated.

Biological annotation reduces the black-box character of the composite but does not prove a common harmful direction or causal pathway. Support-only NEFL adjustment did not materially attenuate the association, yet this does not establish an NfL-independent mechanism or separation from neuronal-injury biology.

NEMPI may complement canonical PD and AD biomarker contexts. SAA, DAT-SPECT, CSF AD biomarkers, and genetic context capture major biological dimensions of PD heterogeneity, but they do not fully summarize broad cognitive-motor progression context. NEMPI may help frame future biomarker-enriched progression studies while remaining distinct from a patient-level decision rule. This positioning is aligned with recent plasma and fluid-biomarker studies showing that multi-protein panels can provide translational information for PD risk, staging, and stratification, while still requiring independent validation before applied use (11–13, 19, 20).

Robustness analyses retained both favorable and cautionary results. First observed, 5-year, discrete-time, age-residualized, stabilized-IPW, technical-context, extended-confounder, and preprocessing analyses were generally consistent. In contrast, persistent/irreversible abnormality had a confidence interval crossing 1, consecutive and GBA/LRRK2 models had events per parameter below 10, fixed age strata were underpowered, the OFF medication-adjusted motor model did not converge, and 5-year LEDD-adjusted MDS-UPDRS II estimates were nonsignificant. These findings bound rather than overturn the primary association.

This study has limitations. It is observational and PPMI-centered, and the observational design does not establish causality or clinical prediction. Independent PD cohorts and assay-setting validation are needed for replication, as emphasized across recent PD fluid-biomarker and large-scale proteomic studies (13, 19, 20). Complete-case denominators differed across analyses, subset models had wider uncertainty, and biofluid proteomics cannot establish causal direction. Multiple imputation was not triggered because ordinary-covariate loss was 0.19%, below the prespecified 5% trigger. Included and excluded candidates differed most in direct-z NEMPI availability and cognitive follow-up density, with smaller imbalances in education, SAA availability, and sex; age and PD duration were closely balanced. The two largest differences directly reflected the analytic requirements for exposure availability and longitudinal outcome ascertainment. Stabilized inverse-probability weighting reduced the maximum absolute SMD between the weighted included sample and the full candidate set to 0.042 and preserved the association direction and magnitude. However, an optimizer precision-loss warning remained, and the analysis does not show that selection bias was removed. Participants in the higher descriptive NEMPI tertile were older, raising the possibility that NEMPI may partly capture aging-related systemic inflammatory or proteomic context. Age was included in the primary Cox model, and age-residualized sensitivity remained supportive, but residual confounding by aging biology, systemic inflammation, comorbidity, medication, body composition, or unmeasured peripheral inflammatory state cannot be excluded. PPMI participants are research volunteers with early PD, relatively high study adherence, and high educational attainment, which may limit generalizability to broader clinical PD populations. Sensitivity models incorporating body mass index, comorbidity burden, anti-inflammatory medication, and preanalytic variables produced similar NEMPI estimates, but residual confounding remains. Smoking was conditional only; renal function, total protein, and sample loading were unavailable, and no proxy variables were constructed. MDS-UPDRS III analyses were secondary motor-context analyses. Explicit ON-state and combined medication-adjusted models converged, whereas the OFF-state medication-adjusted model was singular and provided no reliable replacement estimate. NuLISA NPQ values were platform-normalized, and the direct NPQ and log1p implementations were highly concordant, but assay-setting validation remains necessary. Because NEMPI was standardized within the PPMI analysis set, external application would require a fixed marker whitelist, module structure, preprocessing rule, and centering/scaling parameters rather than redefining the score separately in each cohort. SITE and dominant plate were not missing, and collapsed fixed-effect or cluster-robust sensitivity analyses were supportive; however, uncollapsed SITE and plate variables contained many sparse levels, leaving residual technical-context limitations. Two death-positive participants lacked reliable dates and were not imputed. Among 519 reliable-date participants, no competing death occurred during the cognitive risk window; Fine-Gray was therefore numerically equivalent to the cause-specific model and provided no independent competing-event information. The results do not support a patient-level decision instrument, decision threshold, scoring chart, or individual-level classifier.

Further limitations include research-cohort generalizability, complete-case and follow-up selection, sparse site/plate levels, within-cohort standardization, and lack of external validation. In the CSF AD-biomarker subset, CSF total tau had VIF 16.5, which may reduce precision in that subset but does not affect the primary model. Consecutive-abnormality and GBA/LRRK2 analyses had events per parameter of 8.6 and 8.0, respectively, and fixed age strata also had fewer than 10 events per parameter. The absence of reliable renal-function, total-protein, and sample-loading variables prevents direct assessment of those technical or physiological contexts.

## Conclusions

In baseline cognitively normal PPMI participants with PD, higher NEMPI was associated with the first observed cognitive-status abnormality and modest longitudinal cognitive-motor worsening. Same-sample model fit improved, but incremental discrimination was limited. The results support an abundance-based, neuroimmune-enriched multibiofluid proteomic association marker for group-level progression context, not a patient-level prediction tool, cognition-specific biomarker, clinical threshold, or causal mechanism. Independent-cohort replication and assay-setting validation remain necessary.

## Data Availability

Participant-level data analyzed in this study were obtained on 2026-05-31 from the Parkinson’s Progression Markers Initiative (PPMI) database (https://www.ppmi-info.org/access-data-specimens/download-data), RRID:SCR_006431. For up-to-date information on the study, visit http://www.ppmi-info.org. PPMI data are available to qualified investigators through the PPMI data access process and data-use agreement. Raw participant-level data are not redistributed with this manuscript. Derived summary tables, figure source data, and the Supplementary Data 1 marker-annotation workbook are provided with the submitted materials.
