# Supplementary material for: A neuroimmune-enriched multibiofluid proteomic index associated with cognitive-motor progression in early Parkinson’s disease: a longitudinal PPMI cohort study
Source: Front Immunol. 2026 Sep 14;17:1928940. doi: 10.3389/fimmu.2026.1928940 (PMC13616776; doi:10.3389/fimmu.2026.1928940)
Supplement: Supplementary file 1 [file DataSheet1.docx]

**Supplementary Materials**

This file contains reproducibility details, additional sensitivity and secondary analyses, 17 supplementary tables, and 3 supplementary figures. All analyses preserve the fixed 718-marker whitelist, four modules, equal weights, technical abundance directions, primary outcome, and primary model hierarchy.

**Supplementary Methods**

**Cohort and analysis hierarchy**

The candidate analysis set contained 561 baseline cognitively normal participants with nonmissing baseline visit order. The descriptive set contained 522 participants, and the primary same-sample Cox comparison contained 521 participants with 173 first observed cognitive-status abnormalities. The Base model included age, sex, education, Parkinson's disease duration, baseline MoCA, alpha-synuclein seed amplification assay status, DAT mean striatal specific binding ratio, and APOE epsilon4 carriage; the Extended model added direct-z NEMPI. All additional analyses were secondary, sensitivity, exploratory, or diagnostic and did not change the primary hierarchy.

**Marker eligibility and technical handling**

The pre-outcome marker-eligibility algorithm identified support-only rows before general eligibility assessment. Numeric baseline observations were aligned within the baseline-era visit-order window. Missingness was calculated as one minus the number of unique candidates with a numeric marker value divided by 561. Rows with no numeric values or fewer than 30 numeric participants were unusable; missingness greater than 0.80 defined high missingness. A later greater-than-0.40 branch and a duplicate-ambiguity branch were available but were not triggered. The resulting flow was 11,681 panel-by-biofluid rows to 718 eligible primary markers after 10,906 high-missingness, 53 support-only, and 4 unusable rows. No additional rows were excluded by the 0.40, fewer-than-30, or duplicate-ambiguity branches. Duplicate participant-marker groups were summarized by the median; all 357 groups contained two values, so the median equaled the arithmetic mean. Numeric conversion failures became missing. Platform WARN records were retained. Tukey 1.5-IQR outliers were counted only. No project-level LOD, LLOQ, or ULOQ substitution, deletion, or winsorization was applied.

**NEMPI formulas, coverage, direction, and annotation**

For the primary reporting implementation, direct marker z was calculated as (vendor NPQ minus the marker mean) divided by the marker sample standard deviation, with marker means and sample standard deviations computed from observed values among candidate participants and the sample standard deviation calculated using an n−1 denominator. Vendor NPQ is platform-normalized and log2-scale; it is not a raw read count. The log1p marker-z implementation was examined as a preprocessing sensitivity analysis: nonnegative values used ln(1+NPQ) before within-marker z-standardization; zero remained zero and missing remained missing. All 718 eligible markers were nonnegative. Both implementations used the identical whitelist, modules, technical directions, and equal weights. Within each module, available marker z scores were averaged equally; available module scores were then averaged equally, and the raw overall score was standardized among 557 score-available participants. There was no minimum marker or module coverage threshold: 551 participants had four modules, 6 had two modules, and 4 had none. Higher abundance was retained as a technical direction without outcome-driven flipping and is not a statement that every protein is biologically harmful.

**Cognitive-state outcomes**

First observed abnormality was the first follow-up COGSTATE value of 2 or 3. Consecutive abnormality required two consecutive observed abnormal visits and was timed at the first visit in that sequence. Persistent/irreversible abnormality required that every observed visit after the first abnormal visit remained abnormal. Recovery was any later COGSTATE=1 after the primary event, and recurrent abnormality was a subsequent COGSTATE=2 or 3 after recovery. First PD-MCI, first dementia, and PD-MCI-to-dementia transitions were enumerated. The person-period complementary log-log model used one row per adjacent cognitive-visit interval, log interval length as an offset, follow-up interval indicators for baseline hazard, and participant-clustered standard errors.

**Age, follow-up, selection, and competing risk**

Age analyses included Pearson and Spearman correlations, an age-by-NEMPI interaction, age-residualized NEMPI, and fixed strata of <60, 60-69, and >=70 years with continuous age retained in each stratum. Follow-up analyses included full Cox follow-up, five-year administrative censoring, and linear, five-year restricted, natural-cubic-spline (3 degrees of freedom), and fixed five-year piecewise time forms for MoCA and MDS-UPDRS II/III. Included-versus-excluded comparisons used absolute standardized mean differences for age, sex, education, PD duration, cognitive follow-up count, minimum visit-order difference, and availability of SAA, DAT, and direct-z NEMPI. Post-weighting balance compared the weighted included sample with the full 561-participant candidate set. Stabilized inclusion IPW was truncated at the first and 99th percentiles; robust standard errors, balance, effective sample size, positivity, and warnings were retained. Multiple imputation was planned only if ordinary covariate loss exceeded 5% and was not triggered. Death dates were adjudicated from prespecified PPMI sources. Undated deaths were neither imputed nor replaced by last follow-up. Cause-specific and Fine-Gray analyses used only reliable-date participants.

**Motor state, LEDD, and extended confounding**

Explicit OFF and ON MDS-UPDRS III records were defined from PDSTATE and unambiguous OFFEXAM/ONEXAM flags. Contradictory or unstated records were excluded from state-specific models. Same-participant, same-visit, same-state duplicates were summarized by the median; ON and OFF records were never averaged. Medication-adjusted state-specific models added exact-visit time-varying LEDD and treatment status; the combined model also included state. Baseline and time-varying LEDD sensitivities were separately fitted for MDS-UPDRS II over full and five-year follow-up. Prespecified one-at-a-time Cox sensitivities used baseline BMI, active comorbidity burden with cardiometabolic and systemic inflammatory/autoimmune indicators, baseline active systemic anti-inflammatory medication, or fasting status, collection-to-processing time, processing-to-freezing time, and available plasma volume. Collinear preanalytic variables were not combined. Smoking was descriptive/conditional only. Renal function, total protein, and sample loading were unavailable and no proxies were created.

**MoCA-derived item-sum candidates**

The labels executive-attention and memory refer only to prespecified sums of constituent MoCA items available for the supportive analysis. A score required all constituent items; otherwise the item sum was missing. These measures are not validated domain composites, may share measurement information with the PPMI COGSTATE assessment, and cannot establish executive-function or memory specificity. They are retained as supportive item-sum candidates only.

**Supplementary Results**

Adding NEMPI improved likelihood-based fit in the same sample but produced only a 0.0032 C-index increment with a bootstrap confidence interval crossing 0. The consecutive-abnormality association was stronger but EPP was 8.6; the persistent/irreversible confidence interval crossed 1 (P=0.086). Five-year, discrete-time, age-residualized, stabilized-IPW, and measured confounder/preanalytic estimates remained similar in direction, while fixed age strata were underpowered. Among the 561 candidates, 521 were included and 40 were excluded from the primary same-sample analysis. The largest absolute included-versus-excluded SMDs were 0.471 for direct-z NEMPI availability and 0.406 for cognitive follow-up count. Education, SAA availability, and sex had absolute SMDs of 0.249, 0.226, and 0.164, respectively, whereas age and PD duration were closely balanced. After stabilized IPW, the maximum absolute SMD between the weighted included sample and the full candidate set was 0.042, the effective sample size was 520.1, and no clear positivity violation was identified. The weighted Cox estimate remained similar but retained an optimizer precision-loss warning. No competing death occurred within the observed cognitive risk window, so Fine-Gray added no independent event information. Technical-context sensitivity estimates were HR 1.287 (95% CI 1.069-1.549; P=0.008) with SITE-cluster-robust standard errors, HR 1.238 (95% CI 1.054-1.453; P=0.009) with collapsed SITE fixed effects, and HR 1.284 (95% CI 1.099-1.499; P=0.002) with collapsed dominant-plate fixed effects.

For motor context, explicit OFF and ON unadjusted trajectories were positive. ON-state and combined medication-adjusted models converged; the OFF medication-adjusted model was singular/nonconvergent under each prespecified random-effects simplification. MDS-UPDRS II baseline- and time-varying-LEDD full-follow-up estimates were positive, but both corresponding five-year estimates were nonsignificant. Five-year MoCA, MDS-UPDRS II, and OFF-state MDS-UPDRS III group-average translations were below published MCID ranges.

**Supplementary Tables**

**Supplementary Table S1. Marker-eligibility flow and implemented quality-control branches.**

| **Step / branch** | **Executable rule** | **Rows affected** | **Actual effect on 718-marker set** | **Implementation status** |
| --- | --- | --- | --- | --- |
| Starting rows | Unique panel-by-biofluid marker rows | 11,681 | Starting denominator | Applied |
| Support-only priority | Support-only role assigned before general eligibility | 53 | Excluded from primary NEMPI | Applied |
| Unusable numeric data | No numeric baseline value or n<30 | 4 | All four had n=0; n<30 caused no additional exclusion | Applied; branch effect verified |
| High missingness | Missingness >0.80 | 10,906 | All high-missingness rows were 0.8556-0.9376 | Applied |
| Duplicate ambiguity | duplicate_count > n_subjects_with_rows | 0 | No row excluded | Available; not triggered |
| Secondary missingness branch | Missingness >0.40 after earlier branches | 0 | No row occupied 0.40-0.80 after prior branches | Available; not triggered |
| Primary eligible | Else eligible | 718 | Primary-marker whitelist | Applied |

Missingness denominator was 561 candidates with nonmissing baseline visit order. Rules were implemented before outcome modeling and did not use outcome information. The 0.40, n<30, and duplicate-ambiguity branches had no additional effect. Abbreviations: NEMPI, Neuroimmune-Enriched Multibiofluid Proteomic Index; QC, quality control.

**Supplementary Table S2. NEMPI preprocessing, aggregation, coverage, and direction rules.**

| **Component** | **Implemented rule** | **Observed data consequence** |
| --- | --- | --- |
| Direct marker z | (NPQ-mean)/sample SD among observed candidate participants; sample standard deviation calculated using an n−1 denominator | 718 markers; each retained marker had 554 nonmissing values |
| log1p sensitivity | ln(1+NPQ) when marker minimum >=0; otherwise identity; then marker z | All 718 markers were nonnegative |
| Missing values | Remain missing; aggregation skips missing values | No imputation of NEMPI inputs |
| CSF CNS module | Equal mean of 114 marker z scores | Source-context module |
| CSF inflammation module | Equal mean of 243 marker z scores | Source-context module |
| Plasma CNS module | Equal mean of 116 marker z scores | Source-context module |
| Plasma inflammation module | Equal mean of 245 marker z scores | Source-context module |
| Overall score | Equal mean of available module scores, then z across 557 score-available participants | 551 had 4 modules; 6 had 2; 4 had no score |
| Direction | Higher measured abundance; no outcome-driven flip | Technical direction only, not a harmful-protein claim |
| Outliers | Tukey 1.5-IQR counts only | No deletion or winsorization |
| Assay limits | No project-level LOD/LLOQ/ULOQ substitution | Vendor NPQ retained; numeric failures became missing |

NPQ is vendor-supplied platform-normalized log2-scale normalized protein quantification. Abbreviations: CNS, central nervous system; CSF, cerebrospinal fluid; IQR, interquartile range; LLOQ/LOD/ULOQ, lower limit of quantification/limit of detection/upper limit of quantification; NPQ, normalized protein quantification; NEMPI, Neuroimmune-Enriched Multibiofluid Proteomic Index; SD, standard deviation.

**Supplementary Table S3. Cognitive-state transitions and Cox estimates.**

| **Outcome / transition** | **N** | **Events** | **Median time, years (IQR)** | **HR (95% CI)** | **P value** | **Caution / origin** |
| --- | --- | --- | --- | --- | --- | --- |
| First observed abnormality | 521 | 173 | 3.083 (1.919-5.999) | 1.287 (1.102-1.502) | 0.001 | Primary; baseline |
| Consecutive abnormality | 521 | 77 | 3.915 (2.001-6.998) | 1.525 (1.205-1.931) | <0.001 | EPP=8.6; baseline |
| Persistent/irreversible abnormality | 521 | 115 | 5.084 (2.999-8.085) | 1.182 (0.977-1.431) | 0.086 | 95% CI crosses 1; baseline |
| First PD-MCI | 521 | 171 | 3.083 (1.919-5.999) | Descriptive | - | Baseline |
| First dementia | 521 | 23 | 6.754 (5.125-7.123) | Descriptive | - | Baseline |
| Recovery | 521 | 103 | 1.081 (0.917-1.916) | Descriptive | - | From first abnormality |
| Recurrent abnormality | 521 | 56 | 1.251 (0.999-2.163) | Descriptive | - | From recovery |
| PD-MCI to dementia | 521 | 19 | 4.000 (2.999-5.084) | Descriptive | - | From first PD-MCI |

Hazard ratios are per 1 SD higher direct-z NEMPI using the prespecified covariates. Descriptive transitions were not promoted to primary analyses. Abbreviations: CI, confidence interval; EPP, events per parameter; HR, hazard ratio; IQR, interquartile range; NEMPI, Neuroimmune-Enriched Multibiofluid Proteomic Index; PD-MCI, Parkinson's disease mild cognitive impairment; SD, standard deviation.

**Supplementary Table S4. Incremental model fit, discrete-time survival, and five-year Cox results.**

| **Analysis** | **N** | **Events / intervals** | **NEMPI HR (95% CI)** | **P value** | **AIC** | **BIC** | **C-index / change** |
| --- | --- | --- | --- | --- | --- | --- | --- |
| Base Cox | 521 | 173 | - | - | 1797.93 | 1831.98 | 0.6731 |
| Extended Cox | 521 | 173 | 1.287 (1.102-1.502) | 0.001 | 1789.80 | 1828.10 | 0.6764 |
| Extended minus Base | 521 | 173 | LRT=10.13 | 0.0015 | -8.13 | -3.88 | Delta 0.0032 (-0.0093 to 0.0239) |
| Complementary log-log | 520 | 173 / 2,157 | 1.304 (1.109-1.534) | 0.001 | - | - | Participant-clustered SE |
| Five-year administrative Cox | 521 | 122 | 1.274 (1.051-1.543) | 0.013 | - | - | Sensitivity |

The C-index change confidence interval used 1,000 participant-level bootstrap replicates and crossed 0. The result supports fit improvement with limited incremental discrimination. Abbreviations: AIC, Akaike information criterion; BIC, Bayesian information criterion; CI, confidence interval; HR, hazard ratio; LRT, likelihood-ratio test; NEMPI, Neuroimmune-Enriched Multibiofluid Proteomic Index; SE, standard error.

**Supplementary Table S5. Age association, interaction, and fixed strata.**

| **Analysis** | **N** | **Events** | **Estimate (95% CI)** | **P value** | **EPP / interpretation** |
| --- | --- | --- | --- | --- | --- |
| Pearson NEMPI-age correlation | 557 | - | r=0.384 | <0.001 | Correlation |
| Spearman NEMPI-age correlation | 557 | - | rho=0.399 | <0.001 | Correlation |
| Age x NEMPI interaction | 521 | 173 | HR=0.999 (0.983-1.016) | 0.924 | No interaction evidence |
| Age-residualized NEMPI | 521 | 173 | HR=1.259 (1.093-1.450) | 0.001 | Sensitivity |
| Age <60 years | 190 | 53 | HR=1.507 (1.143-1.986) | 0.004 | EPP=5.9 |
| Age 60-69 years | 214 | 68 | HR=1.161 (0.901-1.496) | 0.249 | EPP=7.6 |
| Age >=70 years | 117 | 52 | HR=1.397 (0.965-2.023) | 0.076 | EPP=5.8 |

Continuous age remained in every stratum model. Low EPP prevents claims of effect heterogeneity across strata. Abbreviations: CI, confidence interval; EPP, events per parameter; HR, hazard ratio; NEMPI, Neuroimmune-Enriched Multibiofluid Proteomic Index.

**Supplementary Table S6. Included-versus-excluded comparison, selection weighting, competing risk, and descriptive/adjusted risk summaries.**

**Panel A. Included-versus-excluded comparison within the 561-participant candidate set.**

| **Variable** | **Included (N=521)** | **Excluded (N=40)** | **Absolute SMD** |
| --- | --- | --- | --- |
| Age, years | 62.52 | 62.43 | 0.010 |
| Female sex, % | 41.8 | 50.0 | 0.164 |
| Education, years | 16.05 | 15.23 | 0.249 |
| PD duration, years | 1.51 | 1.54 (N=24) | 0.019 |
| Cognitive follow-up visits, mean | 6.96 | 5.53 | 0.406 |
| Minimum visit-order difference | 0 | 0 | 0 |
| SAA available, % | 100.0 | 97.5 | 0.226 |
| DAT available, % | 100.0 | 100.0 (N=37) | 0 |
| Direct-z NEMPI available, % | 100.0 | 90.0 | 0.471 |

**Panel B. Selection weighting, competing risk, and descriptive/adjusted risk summaries.**

| **Component** | **Result** | **Caution / interpretation** |
| --- | --- | --- |
| Candidate-set inclusion | 561 candidates; 521 included; 40 excluded | Comparison used SMD, not P-value screening |
| Post-IPW balance versus the full candidate set | Maximum absolute SMD 0.042 | Balance improved; selection bias not assumed eliminated |
| Stable IPW distribution | Min 0.941; median 0.992; max 1.125 | First/99th percentile truncation |
| IPW effective sample size | 520.1 | No clear positivity violation |
| Weighted Cox | HR 1.287 (1.097-1.508); P=0.002 | Optimizer precision-loss warning retained |
| Multiple imputation | Not triggered | Ordinary covariate loss 1/522=0.19%, below 5% |
| Earlier source cohort | Aggregate 1,159 to 575 to 561 | Individual-level comparison not deterministically recoverable |
| Death-positive participants | 40 total; 38 reliable dates; 2 undated | No date imputation |
| In-window competing deaths | 0 | Fine-Gray added no independent event information |
| Cause-specific Cox | N=519; 173 events; HR 1.317 (1.125-1.542); P<0.001 | Reliable-date sample |
| Fine-Gray | N=519; 173 events; sHR 1.317 (1.120-1.547); P<0.001 | 0 competing events in window |
| KM low, 1 year | Risk 2.3% (0.9-6.1) | Tertiles descriptive only |
| KM low, 3 year | Risk 9.3% (5.7-15.0) | Tertiles descriptive only |
| KM low, 5 year | Risk 28.1% (20.1-38.5) | Tertiles descriptive only |
| KM middle, 1 year | Risk 2.9% (1.2-6.9) | Tertiles descriptive only |
| KM middle, 3 year | Risk 16.1% (11.1-23.2) | Tertiles descriptive only |
| KM middle, 5 year | Risk 24.2% (17.3-33.3) | Tertiles descriptive only |
| KM high, 1 year | Risk 6.4% (3.6-11.3) | Tertiles descriptive only |
| KM high, 3 year | Risk 24.5% (18.4-32.2) | Tertiles descriptive only |
| KM high, 5 year | Risk 48.0% (38.9-57.9) | Tertiles descriptive only |
| Adjusted low, 1 year | Risk 3.0% (1.7-4.4) | Tertiles descriptive only |
| Adjusted low, 3 year | Risk 13.0% (9.7-16.6) | Tertiles descriptive only |
| Adjusted low, 5 year | Risk 26.8% (21.2-32.8) | Tertiles descriptive only |
| Adjusted middle, 1 year | Risk 3.7% (2.2-5.4) | Tertiles descriptive only |
| Adjusted middle, 3 year | Risk 15.8% (12.7-19.3) | Tertiles descriptive only |
| Adjusted middle, 5 year | Risk 31.8% (26.7-36.9) | Tertiles descriptive only |
| Adjusted high, 1 year | Risk 4.7% (2.7-7.0) | Tertiles descriptive only |
| Adjusted high, 3 year | Risk 19.4% (15.2-23.9) | Tertiles descriptive only |
| Adjusted high, 5 year | Risk 37.9% (31.7-45.0) | Tertiles descriptive only |

Values in Panel A are means or percentages as shown. Absolute standardized mean differences, rather than P values, were used to describe included-versus-excluded differences. PD duration was available for 24 excluded candidates, and DAT availability was evaluable in 37 excluded candidates. Post-IPW balance compared the weighted included sample with the full 561-participant candidate set. The largest remaining absolute SMD was 0.042. The weighting analysis retained the optimizer precision-loss warning and cannot establish elimination of selection bias. Adjusted risks used covariate standardization under the Extended model with NEMPI fixed at tertile medians. Tertiles do not define a clinical cutoff. Abbreviations: CI, confidence interval; DAT, dopamine transporter; HR, hazard ratio; IPW, inverse-probability weighting; KM, Kaplan-Meier; NEMPI, Neuroimmune-Enriched Multibiofluid Proteomic Index; PD, Parkinson's disease; SAA, alpha-synuclein seed amplification assay; sHR, subdistribution hazard ratio; SMD, standardized mean difference.

**Supplementary Table S7. Full follow-up, five-year, spline, and piecewise longitudinal time forms.**

| **Outcome** | **Time form** | **N** | **Observations** | **NEMPI x time estimate (95% CI) / joint test** | **P value** | **Model note** |
| --- | --- | --- | --- | --- | --- | --- |
| MoCA | Linear full | 517 | 2,520 | -0.060 (-0.103 to -0.017) | 0.006 | Random intercept + slope |
| MoCA | Linear <=5 years | 517 | 1,803 | -0.089 (-0.151 to -0.026) | 0.006 | Restricted window |
| MoCA | Natural cubic spline df=3 | 517 | 2,520 | Joint Wald | 0.006 | All NEMPI-time bases |
| MoCA | Piecewise at 5 years | 517 | 2,520 | Joint Wald | 0.015 | Fixed breakpoint |
| MDS-UPDRS II | Linear full | 521 | 5,594 | 0.162 (0.062-0.262) | 0.001 | Random intercept + slope |
| MDS-UPDRS II | Linear <=5 years | 521 | 4,209 | 0.094 (-0.024-0.213) | 0.119 | Nonsignificant |
| MDS-UPDRS II | Natural cubic spline df=3 | 521 | 5,594 | Joint Wald | <0.001 | P=5.51e-5 |
| MDS-UPDRS II | Piecewise at 5 years | 521 | 5,594 | Joint Wald | <0.001 | P=1.87e-6 |
| MDS-UPDRS III | Linear full | 521 | 4,475 | 0.324 (0.132-0.516) | 0.001 | State-combined outcome |
| MDS-UPDRS III | Linear <=5 years | 521 | 3,744 | 0.319 (0.050-0.588) | 0.020 | Restricted window |
| MDS-UPDRS III | Natural cubic spline df=3 | 521 | 4,475 | Joint Wald | 0.002 | P=0.00162 |
| MDS-UPDRS III | Piecewise at 5 years | 521 | 4,475 | Joint Wald | <0.001 | P=0.000261 |

Longitudinal outcomes are secondary. The five-year MDS-UPDRS II estimate is explicitly retained as nonsignificant. Abbreviations: CI, confidence interval; df, degrees of freedom; MDS-UPDRS, Movement Disorder Society-Unified Parkinson's Disease Rating Scale; MoCA, Montreal Cognitive Assessment; NEMPI, Neuroimmune-Enriched Multibiofluid Proteomic Index.

**Supplementary Table S8. Explicit ON/OFF MDS-UPDRS III models.**

| **Analysis** | **N** | **Observations** | **Median visits** | **Beta/year (95% CI)** | **P value** | **Convergence / caution** |
| --- | --- | --- | --- | --- | --- | --- |
| Explicit OFF, unadjusted | 433 | 1,951 | 4 | 0.302 (0.014-0.590) | 0.040 | Converged |
| Explicit OFF, medication-adjusted | 415 | 1,492 | - | Not reported | - | Singular/nonconvergent in all prespecified random-effects and optimizer attempts |
| Explicit ON, unadjusted | 482 | 2,928 | - | 0.313 (0.125-0.502) | 0.001 | Converged |
| Explicit ON, medication-adjusted | 466 | 1,992 | - | 0.295 (0.095-0.496) | 0.004 | Converged |
| Combined state, medication-adjusted | 474 | 3,484 | - | 0.299 (0.083-0.515) | 0.007 | Converged; state included |

ON and OFF records were never averaged together. Medication-adjusted models included exact-visit time-varying LEDD and treatment status; the combined model also included state. No substitute OFF estimate was selected after nonconvergence. Abbreviations: CI, confidence interval; LEDD, levodopa equivalent daily dose; MDS-UPDRS, Movement Disorder Society-Unified Parkinson's Disease Rating Scale; OFF/ON, medication examination state.

**Supplementary Table S9. MDS-UPDRS II LEDD sensitivities.**

| **LEDD adjustment** | **Follow-up** | **N** | **Observations** | **Beta/year (95% CI)** | **P value** | **Interpretation** |
| --- | --- | --- | --- | --- | --- | --- |
| Baseline LEDD | Full | 521 | 5,594 | 0.162 (0.062-0.261) | 0.002 | Positive |
| Baseline LEDD | <=5 years | 521 | 4,209 | 0.094 (-0.025-0.213) | 0.120 | Nonsignificant |
| Time-varying LEDD | Full | 521 | 2,867 | 0.150 (0.054-0.246) | 0.002 | Positive |
| Time-varying LEDD | <=5 years | 521 | 2,106 | 0.090 (-0.032-0.213) | 0.149 | Nonsignificant |

The two five-year results are retained as nonsignificant. No nearest-visit carry-forward, interpolation, or imputation of LEDD was used. Abbreviations: CI, confidence interval; LEDD, levodopa equivalent daily dose; MDS-UPDRS, Movement Disorder Society-Unified Parkinson's Disease Rating Scale.

**Supplementary Table S10. Clinical effect-size translations and MCID context.**

| **Outcome** | **Annual difference (95% CI)** | **3-year difference** | **5-year difference (95% CI)** | **% baseline SD at 5 years** | **Published MCID context** | **Evidence** |
| --- | --- | --- | --- | --- | --- | --- |
| MoCA | -0.060 (-0.103 to -0.017) | -0.180 | -0.299 (-0.513 to -0.086) | -11.4% | 1.0-2.05 points; 5-year contrast below | Taghizadeh 2026; DOI 10.1038/s41598-026-56514-5 |
| MDS-UPDRS II | 0.162 (0.062-0.262) | 0.487 | 0.812 (0.312-1.312) | 18.6% | 2.51-3.05 points; 5-year contrast below | Horvath 2017; PMID 28218413; DOI 10.1002/mds.26960 |
| MDS-UPDRS III explicit OFF | 0.302 (0.014-0.590) | 0.905 | 1.509 (0.069-2.948) | 13.3% | 3.25-4.63 points; 5-year contrast below | Horvath 2015; PMID 26578041; DOI 10.1016/j.parkreldis.2015.10.006 |

Translations are group-average linear contrasts per 1 SD higher NEMPI, not patient-level predictions, treatment effects, or thresholds. Abbreviations: CI, confidence interval; DOI, digital object identifier; MCID, minimal clinically important difference; MDS-UPDRS, Movement Disorder Society-Unified Parkinson's Disease Rating Scale; MoCA, Montreal Cognitive Assessment; NEMPI, Neuroimmune-Enriched Multibiofluid Proteomic Index; OFF, explicitly assessed off medication; PMID, PubMed identifier; SD, standard deviation.

**Supplementary Table S11. Extended confounder and preanalytic sensitivities.**

| **Sensitivity** | **N** | **NEMPI HR (95% CI)** | **P value** | **VIF / availability** | **Interpretation** |
| --- | --- | --- | --- | --- | --- |
| Base + BMI | 518 | 1.288 (1.104-1.503) | 0.001 | Acceptable | Similar direction/magnitude |
| Base + comorbidity burden/classes | 521 | 1.250 (1.072-1.458) | 0.004 | Acceptable | Similar; not causal independence |
| Base + active anti-inflammatory medication | 521 | 1.276 (1.090-1.494) | 0.002 | Acceptable | Similar; not causal independence |
| Base + preanalytic variables | 515 | 1.287 (1.101-1.504) | 0.002 | All VIF <2.1 | Similar; measured variables only |
| Smoking | - | Not modeled | - | Descriptive only | No harmonized variable was available for formal modeling |
| Renal function | - | Unavailable | - | No proxy constructed | Not assessed because the variable was unavailable |
| Total protein | - | Unavailable | - | No proxy constructed | Not assessed because the variable was unavailable |
| Sample loading | - | Unavailable | - | No proxy constructed | Not assessed because the variable was unavailable |

Each sensitivity was fitted separately; no result-driven combination was used. Preanalytic variables were fasting status, collection-to-processing time, processing-to-freezing time, and available plasma volume. Abbreviations: BMI, body mass index; CI, confidence interval; HR, hazard ratio; NEMPI, Neuroimmune-Enriched Multibiofluid Proteomic Index; VIF, variance inflation factor.

**Supplementary Table S12. Extended clinical context for Table 1.**

| **Characteristic** | **N available** | **Overall value** |
| --- | --- | --- |
| Follow-up duration, years | 521 | 4.00 (2.17-7.92) |
| Cognitive assessments, count | 521 | 4 (3-7) |
| Baseline MDS-UPDRS II | 519 | 6.75 (4.09) |
| Baseline MDS-UPDRS III explicit OFF | 116 | 24.27 (11.37) |
| Baseline MDS-UPDRS III explicit ON | 129 | 17.74 (9.47) |
| Baseline LEDD | 521 | 0 (0-220) |
| PD treatment, n (%) | 521 | 138 (26.5) |
| BMI, kg/m2 | 518 | 26.46 (4.80) |
| Active comorbidity burden | 521 | 0 (0-3) |
| Active systemic anti-inflammatory medication, n (%) | 521 | 136 (26.1) |
| Any explicit ON record, n (%) | 521 | 482 (92.5) |
| Any explicit OFF record, n (%) | 521 | 433 (83.1) |

Values are mean (SD), median (IQR), or n (%). Abbreviations: BMI, body mass index; IQR, interquartile range; LEDD, levodopa equivalent daily dose; MDS-UPDRS, Movement Disorder Society-Unified Parkinson's Disease Rating Scale; OFF/ON, medication examination state; PD, Parkinson's disease; SD, standard deviation.

**Supplementary Table S13. Prespecified cautions and diagnostic findings.**

| **Issue** | **Observed result** | **Required interpretation** |
| --- | --- | --- |
| Incremental discrimination | Delta C-index 0.0032; bootstrap 95% CI -0.0093 to 0.0239 | No discrimination-improvement claim |
| Persistent/irreversible event | HR 1.182; 95% CI 0.977-1.431; P=0.086 | CI crosses 1 |
| Consecutive event | 77 events; EPP 8.6 | Limited precision |
| GBA/LRRK2 subset | EPP 8.0 | Limited precision |
| Age <60 / 60-69 / >=70 | EPP 5.9 / 7.6 / 5.8 | No subgroup-effect claim |
| CSF AD subset | CSF total tau VIF 16.5 | May reduce subset precision; not a primary-model issue |
| IPW | Optimizer precision-loss warning | Retain estimate with computational caution |
| Competing risk | Zero in-window competing deaths | Fine-Gray adds no independent information |
| OFF medication-adjusted MDS-UPDRS III | Singular/nonconvergent | No reliable replacement estimate |
| Five-year MDS-UPDRS II + baseline LEDD | P=0.120 | Nonsignificant retained |
| Five-year MDS-UPDRS II + time-varying LEDD | P=0.149 | Nonsignificant retained |
| Earlier source cohort | Individual-level linkage unrecoverable | Aggregate flow only |
| Renal/total protein/loading | Unavailable | No proxy variables were constructed |

All cautions were retained without altering the prespecified analysis rules. Abbreviations: AD, Alzheimer's disease; CI, confidence interval; CSF, cerebrospinal fluid; EPP, events per parameter; GBA, glucocerebrosidase; HR, hazard ratio; IPW, inverse-probability weighting; LEDD, levodopa equivalent daily dose; LRRK2, leucine-rich repeat kinase 2; MDS-UPDRS, Movement Disorder Society-Unified Parkinson's Disease Rating Scale; VIF, variance inflation factor.

**Supplementary Table S14. Exploratory source-context module Cox decomposition.**

| **Module** | **N** | **Events** | **HR (95% CI)** | **P value** | **BH-FDR q value** |
| --- | --- | --- | --- | --- | --- |
| CSF CNS disease | 519 | 171 | 1.100 (0.945-1.280) | 0.218 | 0.218 |
| CSF inflammation | 519 | 171 | 1.279 (1.015-1.610) | 0.037 | 0.049 |
| Plasma CNS disease | 518 | 172 | 1.258 (1.080-1.465) | 0.003 | 0.013 |
| Plasma inflammation | 518 | 172 | 1.246 (1.060-1.465) | 0.008 | 0.015 |

These module-level analyses are exploratory and do not identify a single causal driver or alter the equal-weight NEMPI. Abbreviations: BH-FDR, Benjamini-Hochberg false-discovery rate; CI, confidence interval; CNS, central nervous system; CSF, cerebrospinal fluid; HR, hazard ratio; NEMPI, Neuroimmune-Enriched Multibiofluid Proteomic Index.

**Supplementary Table S15. Support-only NEFL-adjusted Cox sensitivities.**

| **Support-only context** | **NEMPI HR (95% CI)** | **P value** | **Interpretation** |
| --- | --- | --- | --- |
| CSF NuLISA NEFL-adjusted | 1.40 (1.16-1.69) | <0.001 | Association not attenuated in sample-matched model |
| Plasma NuLISA NEFL-adjusted | 1.32 (1.12-1.56) | <0.001 | Association not attenuated in sample-matched model |

NEFL was support-only and did not enter the primary NEMPI. These analyses assess measured injury-marker overlap; they do not establish an NfL-independent pathway. Abbreviations: CI, confidence interval; CSF, cerebrospinal fluid; HR, hazard ratio; NEFL/NfL, neurofilament light; NEMPI, Neuroimmune-Enriched Multibiofluid Proteomic Index; NuLISA, Nucleic Acid-Linked Immuno-Sandwich Assay.

**Supplementary Table S16. Biological annotation composition of the 718 primary NEMPI markers.**

| **Annotation category** | **Markers, n** | **Percent of 718** |
| --- | --- | --- |
| Broad proteomic context | 320 | 44.6% |
| Adaptive immune, cytokine or chemokine signaling | 264 | 36.8% |
| CNS-enriched proteomic context | 58 | 8.1% |
| Tissue remodeling, vascular or stress-response biology | 36 | 5.0% |
| Microglial or innate immune activation | 22 | 3.1% |
| Neuroaxonal or synaptic injury support | 12 | 1.7% |
| Astroglial activation and glial stress | 6 | 0.8% |
| Total | 718 | 100.0% |

Immune, glial, and vascular/stress categories together comprised 328 of 718 markers (45.7%). NEMPI is therefore described as neuroimmune-enriched rather than purely neuroimmune. Abbreviations: CNS, central nervous system; NEMPI, Neuroimmune-Enriched Multibiofluid Proteomic Index.

**Supplementary Table S17. Leave-one-module-out and site/plate technical-context sensitivity analyses.**

| **Analysis** | **N** | **Events** | **HR** | **95% CI** | **P value** |
| --- | --- | --- | --- | --- | --- |
| Leave out CSF CNS disease module | 521 | 173 | 1.368 | 1.151-1.627 | <0.001 |
| Leave out CSF inflammation module | 521 | 173 | 1.268 | 1.085-1.481 | 0.003 |
| Leave out plasma CNS disease module | 521 | 173 | 1.254 | 1.068-1.474 | 0.006 |
| Leave out plasma inflammation module | 521 | 173 | 1.266 | 1.078-1.487 | 0.004 |
| SITE-cluster-robust standard errors | 521 | 173 | 1.287 | 1.069-1.549 | 0.008 |
| Collapsed SITE fixed effects | 521 | 173 | 1.238 | 1.054-1.453 | 0.009 |
| Collapsed dominant-plate fixed effects | 521 | 173 | 1.284 | 1.099-1.499 | 0.002 |

Leave-one-module-out hazard ratios are per 1 SD higher reduced index; technical-context hazard ratios are per 1 SD higher direct-z NEMPI. All estimates use the prespecified analyses and do not alter the primary hierarchy. SITE denotes the PPMI study-site identifier. Abbreviations: CI, confidence interval; CNS, central nervous system; CSF, cerebrospinal fluid; HR, hazard ratio; NEMPI, Neuroimmune-Enriched Multibiofluid Proteomic Index; PPMI, Parkinson's Progression Markers Initiative.

**Supplementary Figures**

Supplementary Figure S1. Exploratory module and source-context decomposition. Module-level results are exploratory and do not redefine NEMPI or establish a single causal driver. Abbreviations: CI, confidence interval; CNS, central nervous system; CSF, cerebrospinal fluid; HR, hazard ratio; NEMPI, Neuroimmune-Enriched Multibiofluid Proteomic Index.

Supplementary Figure S2. Support-only NfL-adjusted sensitivity analyses. Sample-matched adjustments assess overlap with measured neuroaxonal-injury context; they do not establish an NfL-independent mechanism. Abbreviations: CI, confidence interval; CSF, cerebrospinal fluid; HR, hazard ratio; NfL, neurofilament light; NEMPI, Neuroimmune-Enriched Multibiofluid Proteomic Index.

**Supplementary Figure S3.** NEMPI-tertile cumulative incidence of first observed cognitive-status abnormality. Panel A shows Kaplan–Meier-derived cumulative incidence curves, calculated as 1−S(t); Panel B shows covariate-standardized cumulative incidence curves from the Extended model. The figure reports 1-, 3-, and 5-year risks with 95% confidence intervals. NEMPI tertiles are used only for visualization and do not define clinical thresholds. Abbreviations: CI, confidence interval; NEMPI, Neuroimmune-Enriched Multibiofluid Proteomic Index.
